# UCP1 Induction during Recruitment of Brown Adipocytes in White Adipose Tissue Is Dependent on Cyclooxygenase Activity

**DOI:** 10.1371/journal.pone.0011391

**Published:** 2010-06-30

**Authors:** Lise Madsen, Lone M. Pedersen, Haldis Haukaas Lillefosse, Even Fjære, Ingeborg Bronstad, Qin Hao, Rasmus K. Petersen, Philip Hallenborg, Tao Ma, Rita De Matteis, Pedro Araujo, Josep Mercader, M. Luisa Bonet, Jacob B. Hansen, Barbara Cannon, Jan Nedergaard, Jun Wang, Saverio Cinti, Peter Voshol, Stein Ove Døskeland, Karsten Kristiansen

**Affiliations:** 1 Department of Biology, University of Copenhagen, Copenhagen, Denmark; 2 National Institute of Nutrition and Seafood Research, Bergen, Norway; 3 Department of Biochemistry and Molecular Biology, University of Southern Denmark, Odense, Denmark; 4 Department of Biomedicine, University of Bergen, Bergen, Norway; 5 Department of Biomolecular Sciences, University of Urbino, Urbino, Italy; 6 Laboratory of Molecular Biology, Nutrition and Biotechnology, Universitat de les Illes Balears, and CIBER de Fisiopatología de la Obesidad y Nutrición (CIBERobn), Palma de Mallorca, Spain; 7 Department of Biomedical Sciences, University of Copenhagen, Copenhagen, Denmark; 8 The Wenner-Gren Institute, Stockholm University, Stockholm, Sweden; 9 BGI-Shenzhen, Shenzhen, China; 10 Department of Molecular Pathology and Innovative Therapies, University of Ancona, Ancona, Italy; 11 Metabolic Research Laboratories, University of Cambridge, Cambridge, United Kingdom; University of Hong Kong, China

## Abstract

**Background:**

The uncoupling protein 1 (UCP1) is a hallmark of brown adipocytes and pivotal for cold- and diet-induced thermogenesis.

**Methodology/Principal Findings:**

Here we report that cyclooxygenase (COX) activity and prostaglandin E_2_ (PGE_2_) are crucially involved in induction of UCP1 expression in inguinal white adipocytes, but not in classic interscapular brown adipocytes. Cold-induced expression of UCP1 in inguinal white adipocytes was repressed in COX2 knockout (KO) mice and by administration of the COX inhibitor indomethacin in wild-type mice. Indomethacin repressed β-adrenergic induction of UCP1 expression in primary inguinal adipocytes. The use of PGE_2_ receptor antagonists implicated EP_4_ as a main PGE_2_ receptor, and injection of the stable PGE_2_ analog (EP_3/4_ agonist) 16,16 dm PGE_2_ induced UCP1 expression in inguinal white adipose tissue. Inhibition of COX activity attenuated diet-induced UCP1 expression and increased energy efficiency and adipose tissue mass in obesity-resistant mice kept at thermoneutrality.

**Conclusions/Significance:**

Our findings provide evidence that induction of UCP1 expression in white adipose tissue, but not in classic interscapular brown adipose tissue is dependent on cyclooxygenase activity. Our results indicate that cyclooxygenase-dependent induction of UCP1 expression in white adipose tissues is important for diet-induced thermogenesis providing support for a surprising role of COX activity in the control of energy balance and obesity development.

## Introduction

The two types of adipose tissues, white (WAT) and brown (BAT), have opposite functions in whole body energy homeostasis. Whereas white adipocytes store excess energy as fat, brown adipocytes contain a large number of mitochondria dedicated to convert fat into heat through uncoupled respiration. The uncoupling of respiration and the resulting heat dissipation depend on the expression of the uncoupling protein 1 (UCP1). UCP1 is an integral membrane protein unique to brown adipocyte mitochondria, where it acts as a proton channel to uncouple oxidative phosphorylation by dissipating the proton gradient across the inner mitochondrial membrane [1]. In mice, an increased content of UCP1 in adipose tissue mitochondria is strongly linked to protection against diet-induced obesity. This is true whether increased UCP1 expression is induced by transgenic expression of UCP1 itself [2]; [3], of forkhead box 2 (FOXC2) [4], of PR domain containing 16 (PRDM16) [5] or by disruption of the RIIβ subunit of protein kinase A [6]; [7], eukaryotic translation initiation factor E4-binding protein 1 (4E-BP1) [8], cell death inducing DFFA like effector A and C (Cidea and Cidec/Fsp27) [9], the p160 coregulator TIF2 [10] or retinoblastoma Rb [11]–[13].

Although it has been estimated that 50 g of brown adipocytes would be sufficient to burn 20% of the daily energy intake [14], BAT has traditionally been considered to be virtually absent and of no physiological relevance in adult humans. This view has recently changed dramatically with the demonstration of functional BAT in adult humans [15]–[19] adding to the observation of brown-like multilocular adipocytes expressing UCP1 interspersed within human WAT [20]–[22]. Actually, UCP1 mRNA has been detected in all adipose tissues in adult humans, and it has been estimated that 1 in 100–200 adipocytes in human intraperitoneal adipose tissue expresses UCP1 [23].

Classic interscapular brown adipocytes and brown-like adipocytes found in WAT depots appear to originate from distinct lineages. Brown pre-adipocytes derived from the interscapular region (iBAT) demonstrate myogenic gene expression [24] and classic brown adipocytes arise from Myf5 expressing progenitors [25]. In contrast, brown-like adipocytes appearing in white adipose tissue by β-adrenergic stimulation (“brite adipocytes”) appear to originate from another lineage, much closer to white adipocytes [26]–[29] and display different molecular markers [30]. Several lines of evidence suggest that the number of brown-like adipocytes in WAT depots might influence whole body energy balance. Increased occurrence of brown-like adipocytes within WAT depots is a feature of mouse strains resistant to dietary obesity, such as the A/J strain [31]; [32], and reduced adiposity associated with aP2-transgenic expression of UCP1 is linked to increased energy dissipation in white, but not interscapular brown, adipose tissue [33]. Human obesity is associated with a reduced expression of UCP1 and other thermogenesis related genes in WAT depots [34]; [35]. Thus, identification of factors controlling induction of UCP1 expression and an increase in the number of brown-like adipocytes in white depots obviously deserves further attention.

It is intriguing that the cold-induced occurrence of brown-like adipocytes and UCP1 requires the presence of the β_3_-adrenoceptor in previously white adipose tissue, but not in interscapular brown adipose tissue [36]. Furthermore, the presence of the β_3_-adrenoceptor is required for full stimulation of energy expenditure and oxygen consumption in white adipose tissue [37].

Adipocytes from lean rats have higher isoprenalin-stimulated prostaglandin E_2_ (PGE_2_) synthesis, than adipocytes from obese Zucker rats [38]. We therefore hypothesized that prostaglandins or related products synthesized by cyclooxygenases (COXs) might be involved in the recruitment of brown adipocytes in white depots. The COXs have previously been implicated in adipogenesis [39]–[41], but no specific role has been assigned. Here, we demonstrate that COX activity is crucially involved in the induction of UCP1 expression in WAT providing further evidence for a role of COXs in the control of energy balance and obesity development. In view of the worldwide epidemic of obesity and associated metabolic disorders it is obviously of importance to identify pathways that can be manipulated genetically or pharmacologically and regulate the induction of UCP1 expression and recruitment of brown-like adipocytes in white adipose tissues.

## Results

### COX1 and COX2 protein expression is upregulated in iWAT during cold treatment

**Figure 1:**
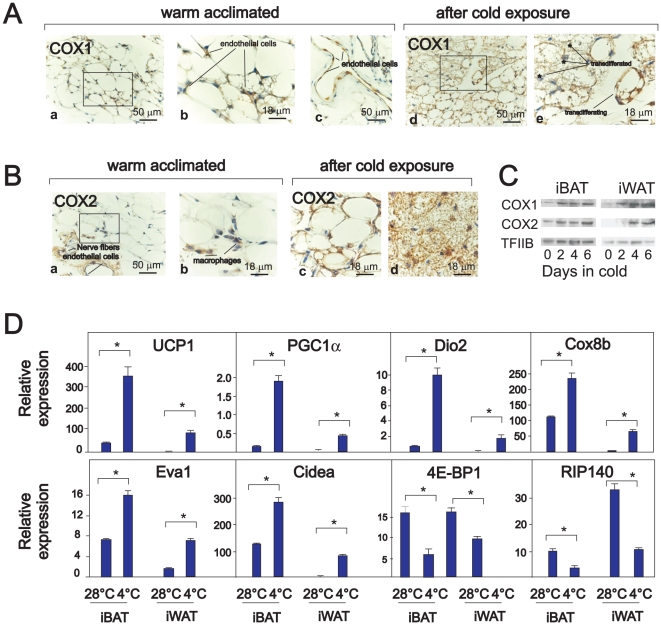
Cold exposure induces COX1 and COX2 expression in iWAT and iBAT. Sv129 mice were warm-acclimated at 28–30°C for 6 days and then transferred to 4–6°C. Samples for cryosections, RNA and protein extractions were prepared from iBAT and iWAT after 2, 4 and 6 days at 4–6°C. **A–B**. Representative COX1 (A) and COX2 (B) immunoreactivity in iWAT from mice kept at 28–30°C for 6 days and after 6 days of cold exposure. **C**. Proteins were isolated from warm-acclimated mice (lane 1) and after 2, 4 and 6 days of cold exposure. COX1 and COX2 expression were determined by Western blotting. **D**. RNA was harvested from iBAT and iWAT from individual mice (n = 4 in each group) that were warm-acclimated or cold-exposed for 6 days. Expressions of genes were measured by RT-qPCR in duplicates and normalized to TBP (TATA box binding protein). The bars represent mean ± standard error. * indicates statistical difference (p<0.05) compared to expression in warm acclimated mice.

When mice are kept at 28°C, close to thermoneutrality, the majority of the adipocytes in the inguinal white adipose depot (iWAT) – the major subcutaneous depot in the mouse – appear as UCP1-negative, spherical unilocular adipocytes [42]. In iWAT from warm-acclimated mice, only endothelial cells and macrophages stained positive for COX1 and COX2, respectively (Figure 1A and B). However, when mice were transferred to a cold environment, the emerging multilocular adipocytes stained positive for COX1 and COX2. In particular, cells that appeared to be in a transition state between uni- and multilocular cells stained strongly (Figure 1A and B). Western blotting demonstrated increased expression of COX1 and COX2 in iWAT, and also in iBAT, after cold exposure (Figure 1C). Real time qPCR analysis verified that genes preferentially expressed in brown *vs.* white adipose tissue, such as UCP1, peroxisome proliferator activated receptor gamma coactivator 1α (PGC1α), type II thyroxine deiodinase (Dio2), cytochrome C oxidase subunit 8b (Cox8b), epithelial V like antigen 1 (Eva1) and Cidea were all highly induced in iWAT upon cold exposure, whereas expression of 4E-BP1 and of nuclear receptor interacting protein 140 (Nrip1/RIP140) was reduced (Figure 1D). Immunohistochemical staining of iBAT from cold-treated mice demonstrated that adipocytes stained positive for COX2, whereas only endothelial cells stained positive for COX1 (Figure S1A). The lack of COX1 and COX2 expression in adipocytes from iBAT in warm-acclimated mice was verified by analysis of protein and RNA isolated from fractionated adipose tissue, in which COX1 and COX2 was detected solely in the stromal vascular fraction (Figure S1B).

### Inhibition of COX activity represses induction of UCP1 expression

**Figure 2:**
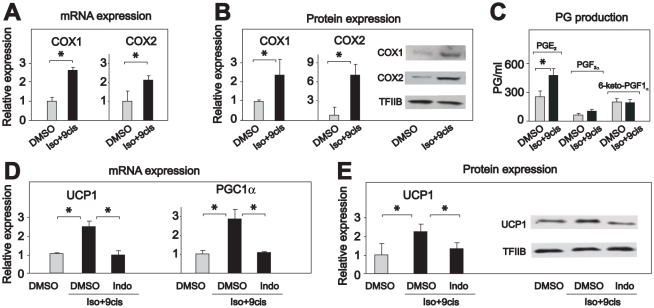
Indomethacin prevents isoproterenol-induced UCP1 expression in *Rb*-negative adipocytes. *Rb*-negative mouse embryo fibroblasts were induced to differentiate as described in experimental procedures. Differentiated cells were treated with vehicle or isoproterenol (100 nM) and 9-cis-retinoic acid (1 µM) for 24 h. Indomethacin (1 µM) was included when indicated in the figure. **A and D.** RNA was isolated and expressions of genes were measured by RT-qPCR in duplicates and normalized to TBP. **C.** The levels of prostaglandin E_2_, F_2α_ and 6-keto-prostaglandin F_1α_ were determined in cell medium using ELISA kits after 24 h. **B and E.** Proteins were harvested and expressions of COX1, COX2 and UCP1 were measured by Western blotting. The bars represent mean ± standard error. The experiments were performed in triplicates and performed 3–5 times. * indicates statistical significant difference (p<0.05).

Differentiated mouse embryo fibroblasts (MEFs) lacking the retinoblastioma (Rb) gene, resemble brown or brown-like adipocytes in demonstrating β-adrenergic induction of UCP1 expression [43]. To achieve a robust induction of UCP1 expression, differentiated Rb^−/−^ MEFs were treated with a combination of isoproterenol and 9-cis retinoic acid [44]. Just as cold exposure increased COX1 and COX2 mRNA and protein levels in brown-like adipocytes (Figure 1), isoproterenol/9-cis retinoic acid treatment increased COX1 and COX2 mRNA and protein expression (Figure 2A and B) in this model system. Upregulation of COX1 and COX2 expression in Rb^−/−^ adipocytes was accompanied by increased production of PGE_2_, the primary prostaglandin produced by mature adipocytes [45]; [46], but not of PGF_2α_ and 6-keto-PGF_1α_ (Figure 2C). This indicates that Rb^−/−^ adipocytes resemble mature adipocytes in producing PGE_2_ as the major prostaglandin species.

To investigate the importance of COX activity for induction of UCP1 expression, we treated differentiated Rb^−/−^ adipocytes with isoproterenol/9-cis retinoic acid in the absence or presence of the general COX inhibitor indomethacin. Indomethacin prevented induction of UCP1 mRNA and protein expression (Figure 2D and E), thus suggesting the intriguing possibility that COX activity is required for induction of UCP1.

**Figure 3:**
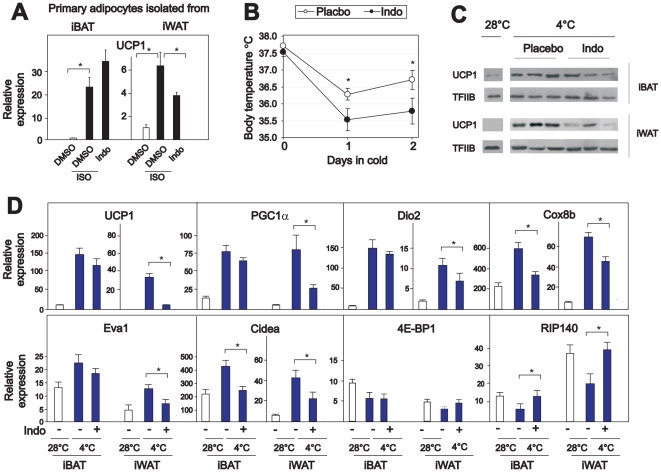
Indomethacin prevents cold-induced UCP1 expression in iWAT. **A.** Stromal vascular fractions (SVFs) were isolated from mouse iWAT and iBAT, cultured and induced to differentiate as described in experimental procedures. Differentiated adipocytes were treated with vehicle or isoproterenol (100 nM) in the absence or presence of indomethacin (1 µM) for 24 h. Expression of UCP1 was measured by RT-qPCR in duplicates and normalized to PPARγ. The bars represent mean ± standard deviation. The experiment was performed in triplicates and repeated 3 times. **B–D.** C57Bl/6 mice were warm-acclimated at 28–30°C for 10 days and then transferred to 4–6°C for 48 h. Mice were injected with vehicle or indomethacin (2.5 mg/kg) 2 h prior transfer to 4°C and thereafter every 12 h. Rectal temperature was measured before the mice were transferred and after 24 and 48 h (B). Protein and RNA extractions were isolated after 48 h. UCP1 expression was measured by Western blotting (C) and expressions of genes were measured by RT-qPCR and normalized to TBP (D). The bars represent mean ± error (n = 5–6). * indicates statistical significant difference (p<0.05).

To examine if COX activity was required also in primary adipocytes, we induced cells from the stromal vascular fraction of iBAT and iWAT to differentiate and then treated the mature adipocytes with the β-adrenergic agonist isoproterenol in the absence and presence of indomethacin. Interestingly, indomethacin inhibited isoproterenol-induced UCP1 expression in cells derived from iWAT but not from iBAT (Figure 3A), indicating that COX activity is required for β-adrenergic induction of UCP1 expression in adipocytes from iWAT, but not in iBAT adipocytes. In keeping with this notion, indomethacin only marginally attenuated induction of UCP1 expression in the WT-1 cell model representing interscapular brown adipocytes (Text S1, Figure S2) [47].

To investigate the role of COX activity during induction of UCP1 expression in iWAT and iBAT *in vivo*, we treated warm-acclimated mice with the COX inhibitor indomethacin and transferred the mice to 4°C. Measurements of rectal temperature revealed that mice treated with indomethacin had slightly, but significantly lower body temperature (Figure 3B). As expected, UCP1 expression was induced in both iBAT and iWAT in vehicle-treated mice (Figure 3C and D). While indomethacin treatment only slightly attenuated cold-induced UCP1 expression in iBAT, it almost completely prevented the induction of UCP1 expression in iWAT (Figure 3C and D). Thus, COX activity appeared to be necessary for cold-induced UCP1 expression in iWAT, but not in iBAT. In addition, indomethacin treatment attenuated cold-induced enhancement of PGC1α, Dio2, Cox8b, Eva1 and Cidea expression in iWAT, while preventing cold-induced repression of RIP140 and 4E-BP1 expression in iWAT (Figure 3D).

### Forced expression of COX2 induces UCP1 expression in Rb^−/−^ adipocytes

**Figure 4:**
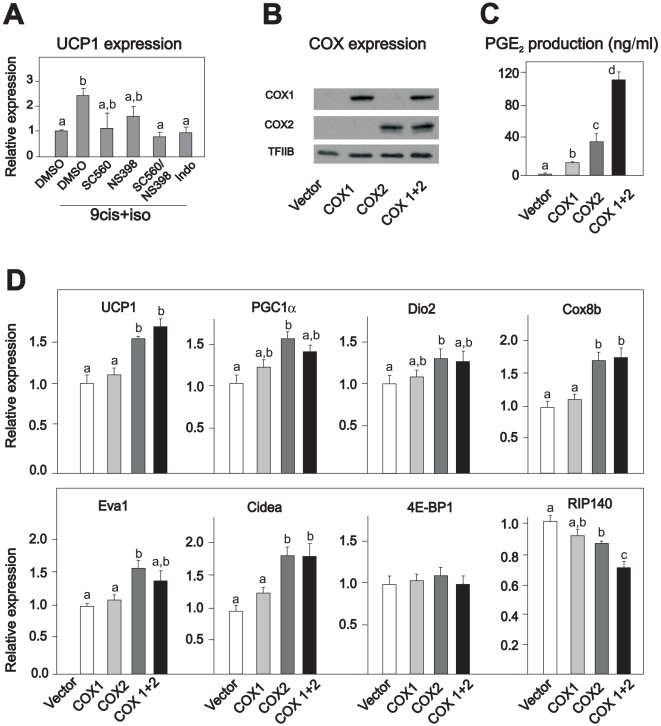
UCP1 expression is induced by forced expression of COX2 alone or in combination with COX1 in cultured cells. **A.** Rb^−/−^ MEFs were induced to differentiate as described in experimental procedures. Differentiated adipocytes were treated with vehicle or isoproterenol (100 nM) and 9-cis-retinoic acid (1 µM) in the absence and presence of the COX1 inhibitor SC560 (50 nM) or the COX2 inhibitor NS398 (5 µM), alone or in combination, or with indomethacin (1 µM), for 24 h. Expression of UCP1 was measured by RT-qPCR. The bars represent mean ± standard error. The experiment was performed in triplicates and repeated 2 times. **B–D.** Rb^−/−^ MEFs were retrovirally transduced with empty vector, vector encoding COX1 or COX2, or both. The transduced cells were selected and induced to differentiate and analyzed for COX1 and COX2 expression by Western blotting (B). PGE_2_ levels were measured in cell media (C). RNA was isolated on day 8 and expressions of genes were measured by RT-qPCR (D). The bars represent mean ± standard error. Different letters indicate statistically significant difference (p<0.05). The experiments were performed in triplicates.

Since indomethacin attenuated β-adrenergically stimulated UCP1 expression in Rb^−/−^ adipocytes and primary inguinal adipocytes, but not in WT-1 cells and primary interscapular brown adipocytes, we again used Rb^−/−^ adipocytes as a model system for “brite” adipocytes. To investigate the relative importance of COX1 and COX2 activities in mediating induction of UCP1 expression in such cells, we treated Rb^−/−^ adipocytes with isoproterenol/9-cis retinoic acid in the absence and presence of selective COX1 and COX2 inhibitors. As shown in Figure 4A, selective inhibition of COX1 and COX2 with SC560 or NS398, respectively, partially prevented UCP1 induction, whereas a combination of these inhibitors or treatment with the non-selective inhibitor indomethacin fully prevented UCP1 induction. Accordingly, activities of both COX1 and COX2 seem necessary for full UCP1 induction.

To further examine the relative importance of COX1 and COX2 for prostaglandin synthesis and UCP1 expression, these enzymes were retrovirally expressed both singly and in combination in Rb^−/−^ MEFs (Figure 4B). The cells were induced to differentiate, and on day 8, the medium was replaced by fresh medium, which was harvested 24 h later and analyzed for PGE_2_ content. The level of PGE_2_ was higher when the cells were transduced with COX2 alone or in combination with COX1, than with COX1 alone (Figure 4C). These results, together with the fact that PGE_2_ formation in adipose tissue in COX2 KO mice is significantly lower than in COX1 KO mice [48], point to COX2 expression as being of major importance for PGE_2_ production. In accordance with this, forced expression of COX1 alone was unable to induce UCP1 expression (Figure 4D). However, UCP1 expression was significantly induced by forced expression of COX2 alone or in combination with COX1 (Figure 4D). Increased expression of UCP1 was accompanied with increased expression of PGC1α, Dio2, Cox8b, Eva1 and Cidea, as well as reduced expression of RIP140, but not 4E-BP1 (Figure 4D).

### Cold-induced UCP1 expression is attenuated in iWAT in COX2 KO mice

**Figure 5:**
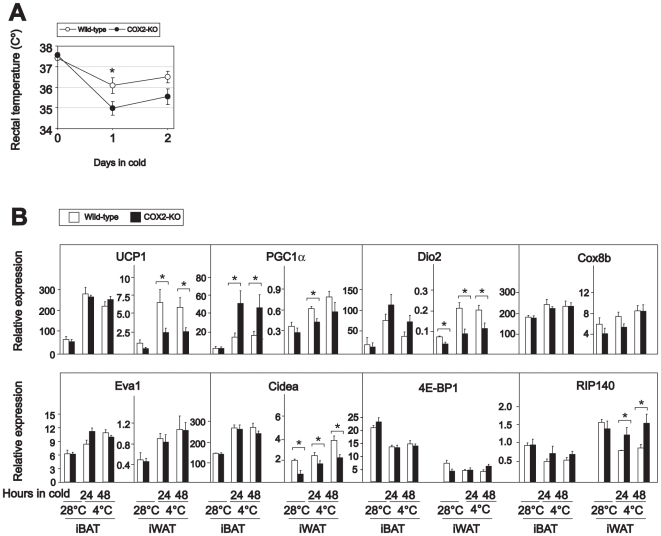
Cold-induced UCP1 expression is attenuated in iWAT in COX2 KO mice. **A B** COX2 KO mice and wild-type littermates were warm-acclimated at 28–30°C for 10 days and then transferred to 4–6°C for 48 h. Rectal temperature was measured before transfer and after 24 and 48 h (A). RNA was extracted and expressions of genes were measured by RT-qPCR (B). The bars represent mean ± standard error (n = 5–6). * indicates statistical significant difference between wild-type and KO mice (p<0.05).

To confirm the importance of COX2 for UCP1 induction in iWAT, wild-type and COX2 KO mice were challenged with a cold environment after warm acclimation. The wild-type mice defended their body temperature better than the COX2 KO mice (Figure 5A). The COX2 KO mice develop severe nephropathy and are susceptible to peritonitis in early life [49]; therefore, KO and wild-type littermates 6 weeks of age were used in this experiment. Unfortunately, we were unable to collect sufficient amounts of iWAT from these young mice to detect UCP1 or COX by Western blotting. However, as expected, cold-induced UCP1 mRNA expression was attenuated in iWAT in COX2 KO mice (Figure 5B). Cold-induced expression of Dio2 and Cidea was also attenuated in iWAT in the COX2 KO mice and PGC1α expression also tended to be attenuated (Figure 5B). Moreover, the cold-induced reduction of RIP140 expression was prevented in the COX2 KO mice (Figure 5B). Expression of Cox8b, Eva1 and 4E BP1 was, however, not significantly different in iWAT from wild-type and COX2 KO mice, suggesting that inhibition of both COX1 and COX2 might be necessary to attenuate cold-induced changes in the expression of these genes. As expected, we observed no differences in UCP1 expression in iBAT in COX2 KO and wild-type mice, and surprisingly, cold-treated COX2 KO mice had significantly higher expression of PGC1α in iBAT than did wild-type mice (Figure 5B).

### PGE_2_ induces UCP1 expression via activation of the EP_3_/EP_4_ receptors

**Figure 6:**
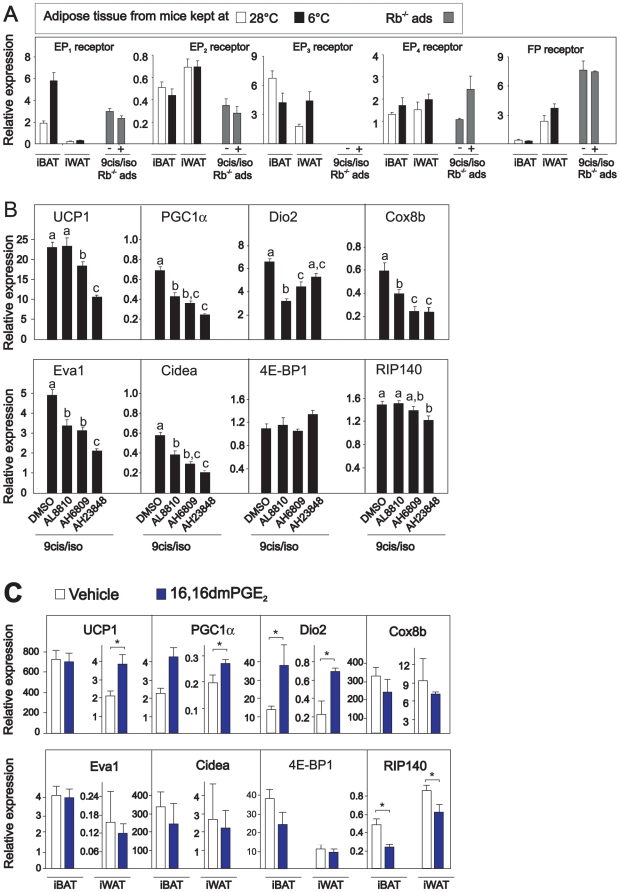
UCP1 expression is attenuated by an EP_4_ receptor antagonist in Rb^−/−^ adipocytes and induced by the EP4 receptor agonist 16,16dmPGE_2_ *in vivo*. **A.** Expressions of EP_1_, EP_2_, EP_3_, EP_4_ and FP receptors were measured by RT-qPCR in iBAT and iWAT isolated from warm- and cold-acclimated mice, and in Rb^−/−^ adipocytes treated with vehicle or isoproterenol (100 nM) and 9-cis-retinoic acid (1 µM). **B.** Rb^−/−^ adipocytes were treated with vehicle or isoproterenol (100 nM) and 9-cis-retinoic acid (1 µM) by RT-qPCR in absence and presence of AL8810, AH6809, or AH23848, which are FP, EP_1_/EP_2_ and EP_4_ receptor antagonists, respectively. Expressions of genes were measured by RT-qPCR. The bars represent mean ± standard error. Different letters indicate statistically significant differences (p<0.05). **C.** C57BL/6J mice were subcutaneously injected with vehicle or 16,16dmPGE_2_ (50 µM/kg) every 12 h for 48 h. Expressions of genes were measured by RT-qPCR. The bars represent mean ± standard error (n = 5). * indicates statistical significant difference between vehicle and 16,16 dmPGE_2_ treated mice (p<0.05).

PGE_2_ is reported to mediate its action by interacting with four subtypes of PGE receptors, the EP_1_, EP_2_, EP_3_ and EP_4_ receptors [50], but may also bind to the prostaglandin F (FP) receptor with an affinity that is only 10–30 fold lower than that of PGF_2α_ [51]. In order to probe the relative importance of these receptors in mediating the possible effect of PGE_2_ on induction of UCP1, expression of the EP and FP receptors was measured in adipose tissue and in Rb^−/−^ adipocytes. All receptors were expressed in both white and brown adipose tissue, whereas no expression of the EP_3_ receptor could be detected in Rb^−/−^ adipocytes (Figure 6A), implying a minor if any role of this receptor in mediating the PGE_2_ response in those cells. Thus, Rb^−/−^ adipocytes were treated with isoproterenol and 9-cis retinoic in the absence or presence of AL8810, AH6809, or AH23848, that are FP-, EP_1_/EP_2_ and EP_4_ receptor antagonists, respectively. Isoproterenol-stimulated UCP1 expression was not affected by the FP receptor antagonist, but slightly attenuated by the EP_1_/EP_2_ receptor antagonist and strongly attenuated by the EP_4_ antagonist (Figure 6B). Reduced expression of UCP1 was accompanied by reduced expression of PGC1α and Cidea (Figure 6B). To verify the importance of PGE_2_ signaling via the EP_4_ receptor with a possible minor contribution by the EP_3_ receptor, mice were injected with an EP_3_/EP_4_ receptor agonist [52], the stable PGE_2_ analogue 16,16-dimethyl-PGE_2_ As predicted, qPCR analysis revealed that UCP1 expression was induced in iWAT, but not in iBAT (Figure 6C). Together, the *in vitro* and *in vivo* results suggest that PGE_2_-induced UCP1 expression at least in part is mediated via the EP_3_/EP_4_ receptors with EP_4_ being the predominant receptor involved.

### Inhibition of COX activity increases adiposity and energy efficiency in obesity resistant Sv129 mice

**Figure 7:**
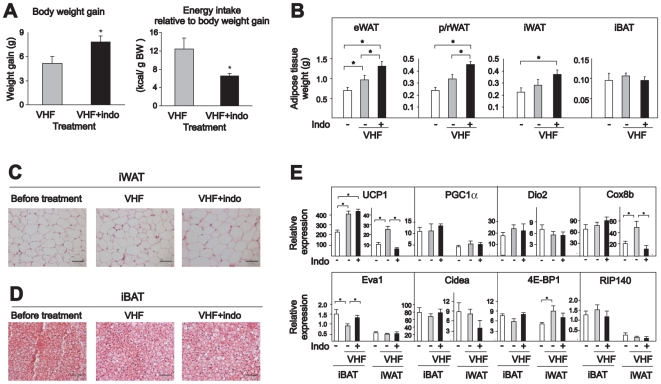
Indomethacin prevents high-fat diet-induced UCP1 expression in iWAT but not iBAT in the obesity-resistant Sv129 mouse strain. Mice were fed a very high-fat diet (VHF) with or without indomethacin supplementation (16 ppm) for 4 weeks at a temperature of 28–30°C. One group of mice was killed before the experiment started. **A.** Body weight gain and energy intake relative to body weight gain. **B.** Different adipose tissue depots were dissected and weighed. **C** and **D**. Representative paraffin-embedded representative sections from iWAT and iBAT were stained with hematoxylin and eosin. The scale bars represent 50 µM. **E.** Expressions of genes in iWAT and iBAT were measured by RT-qPCR. The bars represent mean ± standard error (n = 6). * indicates statistical significant difference (p<0.05) between different groups.

Diet-induced thermogenesis protects several mouse strains against obesity [53]–[55]. Since it appears that the protection against diet-induced obesity is related to increased occurrence of brown-like adipocytes in white depots [56]; [57], we aimed to investigate the hypothesis that indomethacin could also attenuate diet-induced UCP1 expression and thereby increase the propensity for diet-induced obesity in Sv129 mice. Since it was recently demonstrated that UCP1-deficient mice become obese when housed at thermoneutrality [58], we predicted that the most pronounced effect of COX inhibition would be observed for mice kept under thermoneutral conditions. Accordingly, we fed Sv129 mice a very high-fat diet with or without indomethacin supplementation for 4 weeks while keeping the mice at 28–30°C. As demonstrated in Figure 7A, indomethacin supplementation led to a higher weight gain. Energy intake was slightly, but not statistically significantly lower (data not shown). However, energy intake relative to body weight gain was significantly lower in mice that received the indomethacin-supplemented high-fat diet (Figure 7A). Mice fed the diet supplemented with indomethacin also had more WAT in different depots, but not iBAT (Figure 7B). Histological analysis revealed that the adipocytes in both iWAT and iBAT appeared normal, but adipocytes in iWAT in mice fed the high-fat diet supplemented with indomethacin were slightly larger (Figure 7C and D). As expected, feeding mice a high-fat diet lead to augmented expression of UCP1 in both iBAT and iWAT in vehicle-treated mice (Figure 7E). Importantly, diet-induced UCP1 expression was prevented in iWAT, but not in iBAT in the indomethacin-treated mice (Figure 7E). Reduced expression of UCP1 in iWAT in mice fed a high-fat diet supplemented with indomethacin was accompanied with reduced expression of Cox8b. Expression of PGC1α, Dio2, Eva1, Cidea, 4E-BP1 and RIP140 was not affected by inclusion of indomethacin with the high-fat diet (Figure 7E). Collectively, these results underscore the notion that inhibition of COX activity attenuates the acquisition of “brite” adipocytes in white adipose depots with an accompanying increase in feed efficiency leading to accumulation of more adipose tissue. Obviously, other mechanisms may contribute to the increase in feed efficiency, but the lack of “brite” adipocyte recruitment seems a key player.

## Discussion

The unique energy-dissipating ability of UCP1 makes control of its expression and activation potential targets for the development of novel drugs for the treatment of obesity and obesity-associated diseases. Here we present evidence that COX activity and COX-derived PGE_2_ are intimately linked to induction of UCP1 expression in iWAT, but not in iBAT. Thus, cold-induced expression of UCP1 in iWAT was repressed in mice treated with the general COX inhibitor indomethacin, and in COX2 KO mice. Also, injection of a stable analog of the COX2 downstream product PGE_2_, 16,16-dimethyl-PGE_2_, induced UCP1 expression in iWAT. Forced expression of COX2, alone or in combination with COX1, induced UCP1 expression in a cell model resembling inguinal adipocytes. Finally, the inhibition of COX activity not only attenuated diet-induced UCP1 expression in iWAT, but also increased weight gain in Sv129 mice kept at thermoneutrality.

The association between diet-induced thermogenesis and the recruitment of brown adipose tissue was first noted more than 30 years ago and believed to involve the classical brown adipose tissue located in the interscapular region [59]. The anti-obesity role of UCP1 was challenged by the finding that UCP1 KO mice were not obese [60]. However, the recent demonstration that UCP1 ablation *per se* induced obesity when the mice were kept at thermoneutrality [61] clearly indicates that UCP1 is important in diet-induced energy dissipation at thermoneutrality. Our data indicate that inhibition of COX activity increased weight gain and concomitantly attenuated diet-induced UCP1 expression in iWAT, but not iBAT in Sv129 mice kept at thermoneutrality, pointing to a novel role of COX activity in the control of energy balance and the development of obesity. These results are in line with our earlier observation that enhanced cAMP signaling in response to an increased glucagon/insulin ratio led to an increased COX-mediated PGE_2_ production. This was accompanied by increased expression of UCP1 in iWAT, but not in iBAT, and decreased feed efficiency [62]. Although neither COX1 KO nor COX2 KO mice are obese, COX2^+/−^ KO mice have more adipose tissue than wild-type littermates when fed an obesogenic diet [48]. The reason why COX2^+/−^, but not COX2 KO mice were reported to be prone to obesity is not clear. However, these studies were not performed at thermoneutrality [48]. A similar phenomenon is actually seen in GLUT4 KO mice, where the majority of GLUT4^−/+^, but not GLUT4^−/−^ mice develops diabetes [63]. Moreover, release of PGE_2_ from adipose tissue in COX2^+/−^ mice was reported to be reduced compared to adipose tissue from wild-type mice [48] and adipose tissue cultures obtained from obese rats have lower PGE_2_ release rates than cultures from lean rats [38]. In addition, microsomal prostaglandin E synthase1 (mPGES1) expression is reported to be downregulated in iWAT and eWAT in obese mice [64], suggesting a dysregulation of prostaglandin synthesis in obesity.

PGE_2_ mediates its action by interacting with four subtypes of PGE_2_ receptors, the EP_1_, EP_2_, EP_3_ and EP_4_ receptors [50]. Using the Rb^−/−^ brown-like adipocytes, we show that β-adrenergic stimulation of UCP1 expression is attenuated by an EP_4_ receptor antagonist. This combined with our finding that injection of the EP_3_/EP_4_ receptor agonist 16,16-dimethyl-PGE_2_ increased expression of UCP1 in iWAT indicates that the action of PGE_2_ is predominantly mediated via the EP_4_ receptor with a possible minor contribution by the EP_3_ receptor. Most EP_4_ KO mice die shortly after birth, and no adipose tissue phenotype has been reported for the few surviving pups [65]. Similarly, to our knowledge, no adipose tissue phenotype has been reported for the EP_3_ KO mice.

Collectively, our findings strongly indicate that both cold- and diet-induced expression of UCP1 in iWAT, but not in iBAT, requires COX activity and most likely PGE_2_ formation. Furthermore, our results point to differential roles of induction of UCP1 expression in iWAT and iBAT in the context of diet- and cold-induced thermogenesis. We suggest that whereas UCP1 in iWAT plays an important role in protection against obesity, UCP1 in iBAT is essential for temperature adaptation. Upon cold challenge, body temperatures were only slightly lower in wild-type mice treated with indomethacin and in COX2 KO mice compared to non-treated wild-type mice. This is in line with the earlier finding that UCP1 expression is blunted in iWAT, but not in iBAT, in cold-adapted β_3_ adrenoreceptor KO mice [66].

The importance of COX in diet-induced expression of UCP1 in iWAT and diet-induced thermogenesis is underscored by our demonstration that inclusion of the general COX inhibitor indomethacin in the diet augmented high-fat diet-induced obesity in Sv129 mice kept at thermoneutrality irrespectively of UCP1 expression in iBAT. Increased expression of UCP1 in WAT with accompanying increased thermogenic activity coupled with unchanged or even reduced BAT activity has been observed in several genetically modified lean mouse models such as RIP140 [67], Caveolin 1 [68], Fsp27 [69], hormone sensitive lipase [70] and vitamin D receptor [71] KO mice. Further, in RIIβ mice [72], pRb-deficient mice [11]; [73] and in mice overexpressing FOXC2 [74], protection against diet-induced obesity is accompanied by an increased RIα/RIIβ ratio, rendering PKA somewhat more sensitive to cAMP, which is accompanied by an increased occurrence of brown adipocytes in WAT. Last, it should be recalled that in aP2-UCP1 transgenic mice, both endogenous UCP1 expression and respiration are actually reduced in iBAT [75]. UCP1 expression, respiration and total oxidative capacity are, however, strongly induced in WAT and the oxidative capacity of WAT is sufficient for the changes of total energy balance induced by the transgene [76]. In keeping with the earlier notion that i) mouse strains that have more UCP1-expressing adipocytes in their WAT depots are protected against diet-induced obesity [77]; [78] and ii) brown-like multilocular adipocytes expressing UCP1 are detected interspersed within white adipose tissue in humans [20]; [21]; [79], we suggest that factors influencing UCP1 expression in white adipose tissue are of particular importance for the regulation of energy balance and the development of obesity also in humans.

## Materials and Methods

### Ethics Statement

The animal experiments were approved by the Norwegian Animal Health Authorities, ID 819 and 888. Care and handling were in accordance with local institutional recommendations.

### Cell culture, transduction and differentiation

Mouse embryo fibroblasts (MEFs) were prepared from wild-type and *Rb* ^−/−^ embryos [80]. The cells were grown and differentiated in AmnioMax Medium as described earlier [81]. Retrovira expressing pLXSN-hygro, pBabe-puro, pLXSN-COX1 or pBabe-COX2 were harvested from Phoenix–Eco cells, plated at 30–40% confluence in DMEM supplemented with 10% fetal bovine serum, and transductions performed as described [82].

### Isolation of the stromal vascular fraction and adipocytes from mice

The stromal vascular fraction and adipocytes were obtained from iWAT and iBAT dissected from 8-week old C57BL/6J mice as earlier described [83]. Contaminating erythrocytes were eliminated from the stromal-vascular fraction by a wash with sterile distilled water. Cells were plated and induced to differentiate as described [83].

### Cold acclimation experiments

Groups (n = 5–8) of 10-week old male mice were acclimated at a temperature of 28–30°C for at least 1 week and transferred to 4°C for 1, 2, 3 or 6 days. Where relevant, mice were injected with indomethacin (2.5 mg/kg) 2 h prior transfer to 4°C. The mice received a dose of indomethacin every 12 h. Injections were performed subcutaneously from a 0.75 mg/ml solution. Final dose was 5 mg/kg/day. Control mice received vehicle. Animals were housed individually with a 12 h light/dark cycle and free access to pellet food and water. Mice used for immunohistochemical analyses were immediately perfused intracardially with 4% paraformaldehyde. iBAT, iWAT, lung and skin were dissected and frozen for immunohistochemistry on cryosections. For morphology experiments, the mice were immediately perfused with 4% paraformaldehyde in 0.1 M phosphate buffer, pH 7.4 for 5 min. COX2 KO mice (*B6;129P2 Ptgs2 tm1Unc*) and corresponding wild-type littermates were obtained from Taconic. C57BL/6J used in indomethacin experiments were obtained from Møllegård breeding laboratories.

### 16,16dmPGE_2_ injections

Male, C57BL/6J approx 10-weeks old from Møllegård breeding laboratories, Denmark were divided into two groups (n = 5). The mice received a dose of 50 µg/kg 16,16dmPGE_2_ every 12 h for 48 h. Injections were performed subcutaneously and the total dose was 0.1 mg/kg/day. Control mice received vehicle. Animals were housed individually with a 12 h light/dark cycle and free access to pellet food and water.

### High-fat feeding

Male Sv129 mice, 11 weeks old, were obtained from Taconic. The mice were acclimated for 1 week at a temperature of 28–30°C and divided into three groups (n = 6 in each). One group of mice was sacrificed before dietary intervention while the remaining mice were fed a very high-fat diet (ssniff EF R/M acc D12492) with or without indomethacin supplementation (16 ppm) for 4 weeks at a temperature of 28–30°C. Body weight and feed intake were recorded twice a week. Mice were anesthetized using isoflurane, cardiac puncture was performed and mice were killed by cervical dissociation. Tissues were immediately frozen in liquid N_2_.

### Real time qPCR

Total RNA was extracted from cultured cells or mouse tissue using TRIzol (Invitrogen). Reverse transcription and qPCR were performed in duplicates as described earlier [83]. Primer sequences are available on request.

### Western blotting

Preparation of extracts from mouse tissues or whole cell dishes, electrophoresis, blotting, visualization and stripping of membranes were performed as described [84]. Primary antibodies used were goat anti-COX1, goat anti-COX2, rabbit anti-UCP1 and rabbit anti-TFIIB antibodies (Santa Cruz Biotechnology). Secondary antibodies were horseradish peroxidase-conjugated anti-mouse, anti-goat or anti-rabbit antibodies obtained from DAKO.

### Immunohistochemistry

COX1 (M-20; sc 1754) and COX2 (M-19; sc 1747) antibodies were obtained from Santa Cruz Biotechnology, diluted 1∶300 on cryosections and 1∶100 on paraffin-embedded sections (for COX1). Lung [85] and skin [86] were used as positive control for both COX1 and COX2 antibodies.

### Histological analyses

Parts of adipose tissue were fixed in 4% formaldehyde in PB buffer for 24 h, washed in PB, dehydrated in ethanol, embedded in paraffin after 2×10 min xylen treatment. Sections (8 µm thick) of the embedded tissue sections were subjected to standard hematoxylin and eosin staining.

## Supporting Information

Text S1 Experimental.
(0.04 MB DOC)

Figure S1 COX1 and COX2 are mainly expressed in the stromal-vascular fraction of iBAT in warm-acclimated mice. A. Sv129 mice were warm-acclimated at 28–30°C for 6 days and then transferred to 4–6°C. Samples for cryosections were prepared from iBAT and iWAT after four days of cold exposure. Representative COX2 immunoblots in iBAT B. iBAT from warm-acclimated mice was fractionated into SVF and adipocyte fractions, respectively. Expression levels of COX1 and COX2 were determined by RT-qPCR and Western blotting.
(15.01 MB EPS)

Figure S2 Inhibition of COX does not prevent isoproterenol/9-cis-retinoic acid-induced UCP1 expression in WT-1 cells. Differentiated WT-1 cells were treated with a combination of isoproterenol (100 nM) and 9-cis-retinoic acid (1 µM) for 24 h. When included, indomethacin (1 µM) were added 2 h before isoproterenol and 9-cis retinoic acid. UCP1 and PGE1α expressions were determined by RT-qPCR.
(2.44 MB EPS)

## References

[1] Cannon B, Nedergaard J (2004). Brown Adipose Tissue: Function and Physiological Significance.. Physiol Rev.

[2] Kopecky J, Hodny Z, Rossmeisl M, Syrovy I, Kozak LP (1996). Reduction of dietary obesity in aP2-Ucp transgenic mice: physiology and adipose tissue distribution.. Am J Physiol.

[3] Kopecky J, Rossmeisl M, Hodny Z, Syrovy I, Horakova M (1996). Reduction of dietary obesity in aP2-Ucp transgenic mice: mechanism and adipose tissue morphology.. Am J Physiol Endocrinol Metab.

[4] Cederberg A, Gronning LM, Ahren B, Tasken K, Carlsson P (2001). FOXC2 Is a Winged Helix Gene that Counteracts Obesity, Hypertriglyceridemia, and Diet-Induced Insulin Resistance.. Cell.

[5] Seale P, Kajimura S, Yang W, Chin S, Rohas LM (2007). Transcriptional control of brown fat determination by PRDM16.. Cell Metab.

[6] Cummings DE, Brandon EP, Planas JV, Motamed K, Idzerda RL (1996). Genetically lean mice result from targeted disruption of the RII[beta] subunit of protein kinase A.. Nature.

[7] Nolan MA, Sikorski MA, McKnight GS (2004). The role of uncoupling protein 1 in the metabolism and adiposity of RII beta-protein kinase A-deficient mice.. Mol Endocrinol.

[8] Tsukiyama-Kohara K, Poulin F, Kohara M, DeMaria CT, Cheng A (2001). Adipose tissue reduction in mice lacking the translational inhibitor 4E-BP1.. Nat Med.

[9] Zhou Z, Yon Toh S, Chen Z, Guo K, Peng Ng C (2003). Cidea-deficient mice have lean phenotype and are resistant to obesity.. Nat Genet.

[10] Picard F, Gehin M, Annicotte JS, Rocchi S, Champy MF (2002). SRC-1 and TIF2 Control Energy Balance between White and Brown Adipose Tissues.. Cell.

[11] Dali-Youcef N, Mataki C, Coste A, Messaddeq N, Giroud S (2007). Adipose tissue-specific inactivation of the retinoblastoma protein protects against diabesity because of increased energy expenditure.. Proc Natl Acad Sci U S A.

[12] Mercader J, Ribot J, Murano I, Feddersen S, Cinti S (2009). Haploinsufficiency of the retinoblastoma protein gene reduces diet-induced obesity, insulin resistance and hepatosteatosis in mice.. Am J Physiol.

[13] Hansen JB, Jorgensen C, Petersen RK, Hallenborg P, De Matteis R (2004). Retinoblastoma protein functions as a molecular switch determining white versus brown adipocyte differentiation.. Proc Natl Acad Sci U S A.

[14] Cannon B, Nedergaard J (2004). Brown Adipose Tissue: Function and Physiological Significance.. Physiol Rev.

[15] Cypess AM, Lehman S, Williams G, Tal I, Rodman D (2009). Identification and Importance of Brown Adipose Tissue in Adult Humans.. N Engl J Med.

[16] van Marken Lichtenbelt WD, Vanhommerig JW, Smulders NM, Drossaerts JMAF, Kemerink GJ (2009). Cold-Activated Brown Adipose Tissue in Healthy Men.. N Engl J Med.

[17] Virtanen KA, Lidell ME, Orava J, Heglind M, Westergren R (2009). Functional Brown Adipose Tissue in Healthy Adults.. N Engl J Med.

[18] Zingaretti MC, Crosta F, Vitali A, Guerrieri M, Frontini A (2009). The presence of UCP1 demonstrates that metabolically active adipose tissue in the neck of adult humans truly represents brown adipose tissue.. FASEB J.

[19] Saito M, Okamatsu-Ogura Y, Matsushita M, Watanabe K, Yoneshiro T (2009). High incidence of metabolically active brown adipose tissue in healthy adult humans: effects of cold exposure and adiposity.. Diabetes.

[20] Garruti G, Ricquier D (1992). Analysis of uncoupling protein and its mRNA in adipose tissue deposits of adult humans.. Int J Obes Relat Metab Disord.

[21] Lean ME, James WP, Jennings G, Trayhurn P (1986). Brown adipose tissue in patients with phaeochromocytoma.. Int J Obes.

[22] Oberkofler H, Dallinger G, Liu YM, Hell E, Krempler F (1997). Uncoupling protein gene: quantification of expression levels in adipose tissues of obese and non-obese humans.. J Lipid Res.

[23] Oberkofler H, Dallinger G, Liu YM, Hell E, Krempler F (1997). Uncoupling protein gene: quantification of expression levels in adipose tissues of obese and non-obese humans.. J Lipid Res.

[24] Timmons JA, Wennmalm K, Larsson O, Walden TB, Lassmann T (2007). Myogenic gene expression signature establishes that brown and white adipocytes originate from distinct cell lineages.. PNAS.

[25] Seale P, Bjork B, Yang W, Kajimura S, Chin S (2008). PRDM16 controls a brown fat/skeletal muscle switch.. Nature.

[26] Himms-Hagen J, Melnyk A, Zingaretti MC, Ceresi E, Barbatelli G (2000). Multilocular fat cells in WAT of CL-316243-treated rats derive directly from white adipocytes.. Am J Physiol Cell Physiol.

[27] Granneman JG, Burnazi M, Zhu Z, Schwamb LA (2003). White adipose tissue contributes to UCP1-independent thermogenesis.. Am J Physiol Endocrinol Metab.

[28] Cinti S (2009). Transdifferentiation properties of adipocytes in the Adipose Organ.. Am J Physiol Endocrinol Metab: 00183.

[29] Granneman JG, Li P, Zhu Z, Lu Y (2005). Metabolic and cellular plasticity in white adipose tissue I: effects of beta3-adrenergic receptor activation.. Am J Physiol Endocrinol Metab.

[30] Petrovic N, Walden TB, Shabalina IG, Timmons JA, Cannon B (2009). Chronic PPARγ Activation of Epididymally Derived White Adipocyte Cultures Reveals a Population of Thermogenically Competent, UCP1-containing Adipocytes Molecularly Distinct From Classical Brown Adipocytes.. J Biol Chem Dec 22. [Epub ahead of print].

[31] Guerra C, Koza RA, Yamashita H, Walsh K, Kozak LP (1998). Emergence of Brown Adipocytes in White Fat in Mice Is Under Genetic Control. Effects on Body Weight and Adiposity.. J Clin Invest.

[32] Watson PM, Commins SP, Beiler RJ, Hatcher HC, Gettys TW (2000). Differential regulation of leptin expression and function in A/J vs. C57BL/6J mice during diet-induced obesity.. Am J Physiol Endocrinol Metab.

[33] Kopecky J, Rossmeisl M, Hodny Z, Syrovy I, Horakova M (1996). Reduction of dietary obesity in aP2-Ucp transgenic mice: mechanism and adipose tissue morphology.. Am J Physiol Endocrinol Metab.

[34] Oberkofler H, Dallinger G, Liu YM, Hell E, Krempler F (1997). Uncoupling protein gene: quantification of expression levels in adipose tissues of obese and non-obese humans.. J Lipid Res.

[35] Semple RK, Crowley VC, Sewter CP, Laudes M, Christodoulides C (2003). Expression of the thermogenic nuclear hormone receptor coactivator PGC-1[alpha] is reduced in the adipose tissue of morbidly obese subjects.. Int J Obes Relat Metab Disord.

[36] Jimenez M, Barbatelli G, Allevi R, Cinti S, Seydoux J (2003). β_3_ -Adrenoceptor knockout in C57BL/6J mice depresses the occurrence of brown adipocytes in white.. European Journal of Biochemistry.

[37] Grujic D, Susulic VS, Harper ME, Himms-Hagen J, Cunningham BA (1997). beta 3-Adrenergic Receptors on White and Brown Adipocytes Mediate beta 3-Selective Agonist-induced Effects on Energy Expenditure, Insulin Secretion, and Food Intake.. J Biol Chem.

[38] Gaskins HD, Hausman DB, Martin RJ, Hausman GJ (1989). Evidence for abnormal prostaglandin synthesis in obese Zucker rat adipose cell cultures.. J Nutr.

[39] Madsen L, Pedersen LM, Liaset B, Ma T, Petersen RK (2008). cAMP-dependent Signaling Regulates the Adipogenic Effect of n-6 Polyunsaturated Fatty Acids.. J Biol Chem.

[40] Petersen RK, Jorgensen C, Rustan AC, Froyland L, Muller-Decker K (2003). Arachidonic acid-dependent inhibition of adipocyte differentiation requires PKA activity and is associated with sustained expression of cyclooxygenases.. J Lipid Res.

[41] Vassaux G, Gaillard D, Darimont C, Ailhaud G, Negrel R (1992). Differential response of preadipocytes and adipocytes to prostacyclin and prostaglandin E2: physiological implications.. Endocrinology.

[42] Cinti S (2009). Transdifferentiation properties of adipocytes in the Adipose Organ.. Am J Physiol Endocrinol Metab.

[43] Hansen JB, Jorgensen C, Petersen RK, Hallenborg P, De Matteis R (2004). Retinoblastoma protein functions as a molecular switch determining white versus brown adipocyte differentiation.. Proc Natl Acad Sci U S A.

[44] Hansen JB, Jorgensen C, Petersen RK, Hallenborg P, De Matteis R (2004). Retinoblastoma protein functions as a molecular switch determining white versus brown adipocyte differentiation.. Proc Natl Acad Sci U S A.

[45] Hyman BT, Stoll LL, Spector AA (1982). Prostaglandin production by 3T3-L1 cells in culture.. Biochim Biophys Acta.

[46] Madsen L, Pedersen LM, Liaset B, Ma T, Petersen RK (2008). cAMP-dependent Signaling Regulates the Adipogenic Effect of n-6 Polyunsaturated Fatty Acids.. J Biol Chem.

[47] Tseng YH, Kokkotou E, Schulz TJ, Huang TL, Winnay JN (2008). New role of bone morphogenetic protein 7 in brown adipogenesis and energy expenditure.. Nature.

[48] Fain JN, Ballou LR, Bahouth SW (2001). Obesity is induced in mice heterozygous for cyclooxygenase-2.. Prostaglandins Other Lipid Mediat.

[49] Morham SG, Langenbach R, Loftin CD, Tiano HF, Vouloumanos N (1995). Prostaglandin synthase 2 gene disruption causes severe renal pathology in the mouse.. Cell.

[50] Ushikubi F, Hirata M, Narumiya S (1995). Molecular biology of prostanoid receptors: an overview.. J Lipin Mediat.

[51] Abramovitz M, Adam M, Boie Y, Carrière MC, Denis D (2000). The utilization of recombinant prostanoid receptors to determine the affinities and selectivities of prostaglandins and related analogs.. Biochimica et Biophysica Acta.

[52] Kunikata T, Tanaka A, Miyazawa T, Kato S, Takeuchi K (2002). 16,16-Dimethyl Prostaglandin E2 Inhibits Indomethacin-Induced Small Intestinal Lesions Through EP3 and EP4 Receptors.. Digestive Diseases and Sciences.

[53] Koza RA, Hohmann SM, Guerra C, Rossmeisl M, Kozak LP (2000). Synergistic Gene Interactions Control the Induction of the Mitochondrial Uncoupling Protein (Ucp1) Gene in White Fat Tissue.. J Biol Chem.

[54] Guerra C, Koza RA, Yamashita H, Walsh K, Kozak LP (1998). Emergence of Brown Adipocytes in White Fat in Mice Is Under Genetic Control. Effects on Body Weight and Adiposity.. J Clin Invest.

[55] Collins S, Daniel KW, Petro AE, Surwit RS (1997). Strain-Specific Response to β3-Adrenergic Receptor Agonist Treatment of Diet-Induced Obesity in Mice.. Endocrinology.

[56] Guerra C, Koza RA, Yamashita H, Walsh K, Kozak LP (1998). Emergence of Brown Adipocytes in White Fat in Mice Is Under Genetic Control. Effects on Body Weight and Adiposity.. J Clin Invest.

[57] Watson PM, Commins SP, Beiler RJ, Hatcher HC, Gettys TW (2000). Differential regulation of leptin expression and function in A/J vs. C57BL/6J mice during diet-induced obesity.. Am J Physiol Endocrinol Metab.

[58] Feldmann HM, Golozoubova V, Cannon B, Nedergaard J (2009). UCP1 Ablation Induces Obesity and Abolishes Diet-Induced Thermogenesis in Mice Exempt from Thermal Stress by Living at Thermoneutrality.. Cell Metabolism.

[59] Rothwell NJ, Stock MJ (1979). A role for brown adipose tissue in diet-induced thermogenesis.. Nature.

[60] Enerbäck S, Jacobsson A, Simpson EM, Guerra C, Yamashita H (1997). Mice lacking mitochondrial uncoupling protein are cold-sensitive but not obese.. Nature.

[61] Feldmann HM, Golozoubova V, Cannon B, Nedergaard J (2009). UCP1 Ablation Induces Obesity and Abolishes Diet-Induced Thermogenesis in Mice Exempt from Thermal Stress by Living at Thermoneutrality.. Cell Metabolism.

[62] Madsen L, Pedersen LM, Liaset B, Ma T, Petersen RK (2008). cAMP-dependent Signaling Regulates the Adipogenic Effect of n-6 Polyunsaturated Fatty Acids.. J Biol Chem.

[63] Stenbit AE, Tsao TS, Li J, Burcelin R, Geenen DL (1997). GLUT4 heterozygous knockout mice develop muscle insulin resistance and diabetes.. Nat Med.

[64] Hetu PO, Riendeau D (2007). Down-regulation of Microsomal Prostaglandin E2 Synthase-1 in Adipose Tissue by High-fat Feeding.. Obesity Res.

[65] Sakuma Y, Tanaka K, Suda M, Komatsu Y, Yasoda A (2000). Impaired Bone Resorption by Lipopolysaccharide In Vivo in Mice Deficient in the Prostaglandin E Receptor EP4 Subtype.. Infect Immun.

[66] Jimenez M, Barbatelli G, Allevi R, Cinti S, Seydoux J (2003). β_3_ -Adrenoceptor knockout in C57BL/6J mice depresses the occurrence of brown adipocytes in white.. European Journal of Biochemistry.

[67] Leonardsson G, Steel JH, Christian M, Pocock V, Milligan S (2004). Nuclear receptor corepressor RIP140 regulates fat accumulation.. Proc Natl Acad Sci U S A.

[68] Razani B, Combs TP, Wang XB, Frank PG, Park DS (2002). Caveolin-1-deficient mice are lean, resistant to diet-induced obesity, and show hypertriglyceridemia with adipocyte abnormalities.. J Biol Chem.

[69] Toh SY, Gong J, Du G, Li JZ, Yang S (2008). Up-Regulation of Mitochondrial Activity and Acquirement of Brown Adipose Tissue-Like Property in the White Adipose Tissue of Fsp27 Deficient Mice.. PLoS ONE.

[70] Ström K, Hansson O, Lucas S, Nevsten P, Fernandez C (2008). Attainment of Brown Adipocyte Features in White Adipocytes of Hormone-Sensitive Lipase Null Mice.. PLoS ONE.

[71] Narvaez CJ, Matthews D, Broun E, Chan M, Welsh J (2009). Lean Phenotype and Resistance to Diet-Induced Obesity in Vitamin D Receptor Knockout Mice Correlates with Induction of Uncoupling Protein-1 in White Adipose Tissue.. Endocrinology.

[72] Cummings DE, Brandon EP, Planas JV, Motamed K, Idzerda RL (1996). Genetically lean mice result from targeted disruption of the RII[beta] subunit of protein kinase A.. Nature.

[73] Hansen JB, Jorgensen C, Petersen RK, Hallenborg P, De Matteis R (2004). Retinoblastoma protein functions as a molecular switch determining white versus brown adipocyte differentiation.. Proc Natl Acad Sci U S A.

[74] Cederberg A, Gronning LM, Ahren B, Tasken K, Carlsson P (2001). FOXC2 Is a Winged Helix Gene that Counteracts Obesity, Hypertriglyceridemia, and Diet-Induced Insulin Resistance.. Cell.

[75] Kopecky J, Rossmeisl M, Hodny Z, Syrovy I, Horakova M (1996). Reduction of dietary obesity in aP2-Ucp transgenic mice: mechanism and adipose tissue morphology.. Am J Physiol Endocrinol Metab.

[76] Kopecky J, Rossmeisl M, Hodny Z, Syrovy I, Horakova M (1996). Reduction of dietary obesity in aP2-Ucp transgenic mice: mechanism and adipose tissue morphology.. Am J Physiol Endocrinol Metab.

[77] Guerra C, Koza RA, Yamashita H, Walsh K, Kozak LP (1998). Emergence of Brown Adipocytes in White Fat in Mice Is Under Genetic Control. Effects on Body Weight and Adiposity.. J Clin Invest.

[78] Watson PM, Commins SP, Beiler RJ, Hatcher HC, Gettys TW (2000). Differential regulation of leptin expression and function in A/J vs. C57BL/6J mice during diet-induced obesity.. Am J Physiol Endocrinol Metab.

[79] Oberkofler H, Dallinger G, Liu YM, Hell E, Krempler F (1997). Uncoupling protein gene: quantification of expression levels in adipose tissues of obese and non-obese humans.. J Lipid Res.

[80] Hansen JB, Jorgensen C, Petersen RK, Hallenborg P, De Matteis R (2004). Retinoblastoma protein functions as a molecular switch determining white versus brown adipocyte differentiation.. Proc Natl Acad Sci U S A.

[81] Hansen JB, Jorgensen C, Petersen RK, Hallenborg P, De Matteis R (2004). Retinoblastoma protein functions as a molecular switch determining white versus brown adipocyte differentiation.. Proc Natl Acad Sci U S A.

[82] Madsen L, Pedersen LM, Liaset B, Ma T, Petersen RK (2008). cAMP-dependent Signaling Regulates the Adipogenic Effect of n-6 Polyunsaturated Fatty Acids.. J Biol Chem.

[83] Madsen L, Petersen RK, Sørensen MB, Jørgensen C, Hallenborg P (2003). Adipocyte differentiation of 3T3-L1 preadipocytes is dependent on lipoxygenase activity during the initial stages of the differentiation process.. Biochem J.

[84] Hansen JB, Jorgensen C, Petersen RK, Hallenborg P, De Matteis R (2004). Retinoblastoma protein functions as a molecular switch determining white versus brown adipocyte differentiation.. Proc Natl Acad Sci U S A.

[85] Bauer AK, Dwyer-Nield LD, Malkinson AM (2000). High cyclooxygenase 1 (COX-1) and cyclooxygenase 2 (COX-2) contents in mouse lung tumors.. Carcinogenesis.

[86] Muller-Decker K, Hirschner W, Marks F, Furstenberger G (2002). The Effects of Cyclooxygenase Isozyme Inhibition onIncisional Wound Healing in Mouse Skin..

