# Supplementary material for: UCP1 Induction during Recruitment of Brown Adipocytes in White Adipose Tissue Is Dependent on Cyclooxygenase Activity
Source: PLoS One. 2010 Jun 30;5(6):e11391. doi: 10.1371/journal.pone.0011391 (PMC2894971; doi:10.1371/journal.pone.0011391)
Supplement: Text S1 — Experimental. (0.04 MB DOC) [file pone.0011391.s001.doc]

**Experimental**

The WT-1 preadipocyte cell line (Tseng, 2004) was kindly provided to Dr J. B. Hansen by Dr. C. Ronald Kahn and was established by immortalisation of primary brown preadipocytes from newborn pups with simian virus 40 large T antigen. WT-1 cells were propagated and differentiated in DMEM supplemented with 10% FBS. For differentiation, 1 day postconfluent cells were treated with DMEM containing 10% FBS, 1 μM dexamethasone, 0.5 mM methylisobutylxanthine, 5 μg/ml insulin and 0.5 μM rosiglitazone. On days 2, 4 and 6, the cells were refed with DMEM containing 10% FBS supplemented with 5 μg/ml insulin and 0.5 μM rosiglitazone. At day 9, cells were treated with a combination of isoproterenol (100 nM) and 9-cis-retinoic acid (1µM) for 24 h. When included, indomethacin (1 µM) was added 2 h before isoproterenol and 9-cis retinoic acid treatment.

**Reference**

1. Tseng, Y. H., Kriauciunas, K. M., Kokkotou, E., and Kahn, C. R. (2004) Differential roles of insulin receptor substrates in brown adipocyte differentiation. *Mol. Cell. Biol.* **24,** 1918 1929
